# The Mitochondrial ATP Synthase/IF1 Axis in Cancer Progression: Targets for Therapeutic Intervention

**DOI:** 10.3390/cancers15153775

**Published:** 2023-07-25

**Authors:** Sonia Domínguez-Zorita, José M. Cuezva

**Affiliations:** 1 Departamento de Biología Molecular, Centro de Biología Molecular Severo Ochoa, Consejo Superior de Investigaciones Científicas-Universidad Autónoma de Madrid (CSIC-UAM), 28049 Madrid, Spain; sonia.dominguez@cbm.csic.es; 2 Centro de Investigación Biomédica en Red de Enfermedades Raras (CIBERER) ISCIII, 28029 Madrid, Spain; 3 Instituto de Investigación Hospital 12 de Octubre, Universidad Autónoma de Madrid, 28041 Madrid, Spain

**Keywords:** cancer, OXPHOS, Warburg effect, mitochondrial ATP synthase, ATPase inhibitory factor 1, metabolic reprogramming, metastasis, RNA binding proteins, mitohormesis, cell death

## Abstract

**Simple Summary:**

Cancer is a global health problem with high personal and economic burden worldwide. The reprogramming of metabolism experienced by carcinomas offers a large set of enzymes that could be exploited to prevent cancer growth and metastasis. This review emphasizes the interplay between mitochondrial ATP synthase and its physiological inhibitor, the ATPase Inhibitory Factor 1 (IF1), in the metabolic reprogramming of OXPHOS in cancer cells to an enhanced glycolytic phenotype. We highlight the cell-type specificity by which the ATP synthase/IF1 axis exerts its activity as a tumor promotor or signals an anti-metastatic phenotype. Moreover, the implication of the ATP synthase/IF1 axis in cell death, and as a promising target for cancer therapy, is also stressed. We think that investigations aimed at characterizing the posttranscriptional mechanisms that regulate the activity of ATP synthase/IF1 axis will provide additional promising biomarkers for effective treatment of the disease.

**Abstract:**

Cancer poses a significant global health problem with profound personal and economic implications on National Health Care Systems. The reprograming of metabolism is a major trait of the cancer phenotype with a clear potential for developing effective therapeutic strategies to combat the disease. Herein, we summarize the relevant role that the mitochondrial ATP synthase and its physiological inhibitor, ATPase Inhibitory Factor 1 (IF1), play in metabolic reprogramming to an enhanced glycolytic phenotype. We stress that the interplay in the ATP synthase/IF1 axis has additional functional roles in signaling mitohormetic programs, pro-oncogenic or anti-metastatic phenotypes depending on the cell type. Moreover, the same axis also participates in cell death resistance of cancer cells by restrained mitochondrial permeability transition pore opening. We emphasize the relevance of the different post-transcriptional mechanisms that regulate the specific expression and activity of ATP synthase/IF1, to stimulate further investigations in the field because of their potential as future targets to treat cancer. In addition, we review recent findings stressing that mitochondria metabolism is the primary altered target in lung adenocarcinomas and that the ATP synthase/IF1 axis of OXPHOS is included in the most significant signature of metastatic disease. Finally, we stress that targeting mitochondrial OXPHOS in pre-clinical mouse models affords a most effective therapeutic strategy in cancer treatment.

## 1. Introduction

Mitochondria are central hubs in cellular physiology integrating cellular metabolism, bioenergetics, the execution of cell death and signaling through different effectors like Ca^2+^, reactive oxygen species (mtROS), mtDNA and different metabolites [1,2,3,4]. Given the fundamental roles played by the organelle, mitochondrial dysfunction is found in many different pathologies that include metabolic syndrome, neurodegeneration, inflammation, cardiovascular and rare diseases, cancer and normal physiological aging [5,6,7,8,9].

In the cancer field, Otto Warburg was first to describe, almost 100 years ago [10], that tumor cells and embryonic tissues have an exacerbated glucose consumption in the presence of oxygen. This observation led him to suggest, in agreement with the Pasteur effect [11], that tumors and embryonic tissues should exhibit an altered mitochondrial function to sustain their abnormal aerobic glycolysis [12]. His hypothesis was heatedly debated and almost abandoned by the outbreak of Molecular Biology and the discovery of oncogenes. In fact, at the beginning of this century, six hallmarks of the cancer phenotype were defined which included self-sufficiency in growth signals, insensitivity to anti-growth signals, limitless replicative potential, tissue invasion and metastasis, sustain angiogenesis and evading apoptosis [13]. However, the reprogramming of energy metabolism was not listed as a cancer hallmark at that stage. It was a decade later that a review by the same authors included two additional hallmarks of cancer: the avoidance of immune surveillance and the reprogramming of metabolism [14,15]. The renascence of Warburg’s postulates in the cancer field was spurred by the implementation of Positron Emission Tomography (PET) in clinical oncology, using the radionuclide ^18^F-dexoxyglucose (^18^FDG) [16,17,18], which represents the bed-side translation of Warburg’s metabolic observation regarding the glucose avidity of tumors [19]. The cancer imaging technique stimulated metabolically oriented “omic” investigations of human carcinomas that opened up new windows of hope in cancer patients for its potential in diagnostic, staging, treatment and follow up of the disease [20,21,22,23,24,25]. In this review, we summarize the role of the mitochondrial ATP synthase and the role of its inhibitor protein, the ATPase Inhibitory Factor 1 (IF1), in metabolic rewiring of cancer and its progression to emphasize the relevance that the engine that controls cellular life and death has in this field of investigation.

## 2. Metabolic Rewiring and Cancer Progression

**Figure 1:**
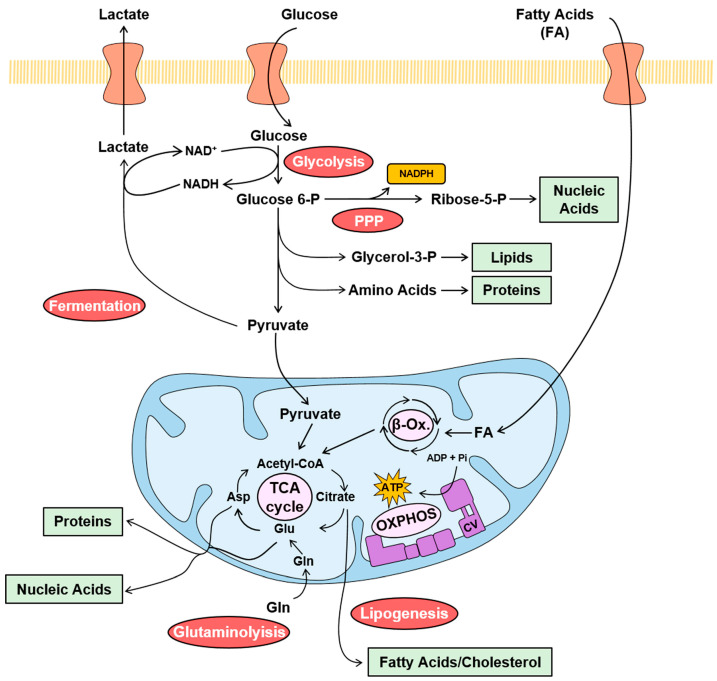
Metabolic pathways in cancer. The color-coded scheme illustrates the major cancer-promoted changes in relevant metabolic pathways observed in human carcinomas. Glycolysis, fermentation, pentose phosphate pathway (PPP), lipogenesis and glutaminolysis are enhanced (red circles) in carcinomas. However, the mitochondrial oxidation of pyruvate, β-oxidation (β-Ox.), tricarboxylic acid (TCA) cycle and oxidative phosphorylation (OXPHOS) are marginally increased (pink circles) or diminished when compared to the activation of metabolic pathway in the cytoplasm. Glucose enters the cell by specific transporters and is metabolized at high rate through glycolysis to provide precursors such as glycerol-3-P and amino acids for lipid and protein synthesis (green box), respectively. Pyruvate is reduced to lactate in fermentation to regenerate NAD^+^ to allow glycolysis to proceed and is exported from the cell. In addition, glucose is catabolized through the PPP to obtain NADPH (orange box), for biosynthetic processes and the maintenance of the cellular redox state, and ribose-5-P, for nucleic acid biosynthesis (green box). In the mitochondria, pyruvate is oxidized to generate acetyl-CoA that feeds the TCA cycle. Fatty acids (FA) oxidation also feeds acetyl-CoA to the TCA cycle. Citrate in the TCA cycle is drained to the cytoplasm for the biosynthesis of FA and cholesterol. Glutaminolysis feeds the TCA cycle with glutamate. The electrons obtained in the oxidation of pyruvate and FA are transferred to the electron transport chain system for the synthesis of the ATP by OXPHOS (purple).

Carcinomas undergo a coordinated reprogramming of their metabolic pathways to satisfy the demand for high energy and for the building blocks that are required to sustain cellular proliferation [21,22,26,27,28,29]. The metabolic reprogramming experienced by cancer is generally known as the Warburg effect, and it is characterized by the sharp increase in the rates of glycolysis and of the fermentation of pyruvate into lactate, which allows the regeneration of NAD^+^ for glycolysis to proceed at high rates (Figure 1). Glycolysis and fermentation do not require oxygen to function. Concurrent with the hyperactivation of glycolysis and fermentation, the oxidation of pyruvate coupled to oxidative phosphorylation (OXPHOS) in mitochondria, which is strictly dependent on the availability of oxygen, is marginally increased and/or partially inhibited in cancer cells, to spare the backbone of carbon skeletons for the synthesis of precursors (Figure 1) [26,29,30,31]. In fact, the citrate formed in the TCA cycle is exported to the cytoplasm for providing the acetyl-CoA that is used for the synthesis of fatty acids and cholesterol (Figure 1) [32]. However, cancer cells replenish carbon skeletons in the TCA cycle in the form of glutamate by increasing the metabolism of glutamine (Figure 1) [33]. Simultaneously, the metabolism of glucose through the pentose phosphate pathway (PPP) is also significantly augmented, to produce the ribose that is used for building nucleotides and producing the NADPH that is used in biosynthetic processes and in the maintenance of the cellular redox state (Figure 1) [34]. Consistent with the reprogramming of metabolism in carcinomas, the sharp increase in different enzymes of glycolysis provide biomarkers that predict a bad patient prognosis [35], whereas, for example, the silencing of glycolytic enzymes in glioblastoma (GBM) xenografts dramatically increase the survival of mice [36]. In contrast, the loss of mitochondrial proteins involved in pyruvate oxidation, β-oxidation and OXPHOS predicts bad patient prognosis [35,37,38,39], supporting the theory that a diminished metabolic activity of the organelle compromises survival. Likewise, the overexpression of enzymes of metabolic pathways that are induced in carcinomas, such as glutamine metabolism [35,40,41] and the biosynthesis of fatty acids [35,42,43,44,45], predicts bad patient prognosis.

The question is, what are the underlying mechanisms that promote metabolic reprogramming in carcinomas? The general idea is that oncogene-driven metabolic changes could explain the metabolic rewiring in cancer, since the activation of oncogenes and/or the inhibition of tumor suppressors (Myc, Akt, p53, HIF-1α, APC/C-Cdh1) certainly affect energy metabolism and could account for changes in metabolic flux [26,46,47,48]. However, the Warburg phenotype is reversible, and could be acquired with or without any oncogenic alteration as demonstrated by adaptation of cancer cells to changing environments [49,50,51,52,53,54,55,56,57,58]. Indeed, the induction of proliferation in normal non-cancer cells or the adaption of cancer cells to different milieu where the tumors develop [50,51,52,59], provide examples in this regard. Overall, we believe that the rewiring of energy metabolism represents a reversible adaptive phenotypic response of the cancer cell to the induction of proliferation, hypoxia and/or other agents of the tumor microenvironment, that results in the hyperstimulation of glycolysis and fermentation affecting mitochondrial OXPHOS in a cell-type specific manner. Energy metabolism provides a therapeutic target with great potential for the treatment of cancer patients, since it is a trait of the disease that is reversible and amenable to modifications by inhibitors of the enzymes that steer metabolism [20,60,61,62,63,64,65,66].

## 3. The Mitochondrial ATP Synthase/IF1 Axis

**Figure 2:**
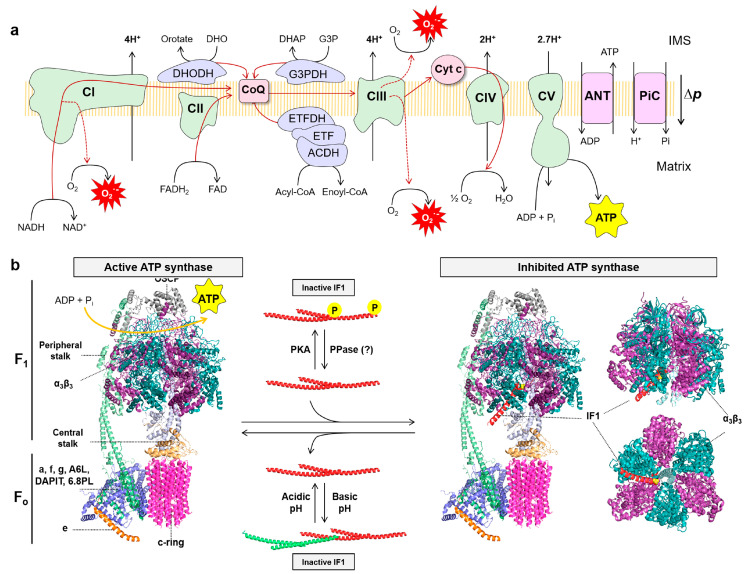
The mitochondrial OXPHOS system: (**a**) The OXPHOS system consists of five protein complexes: complexes I–IV (CI–IV) of the electron transport chain (ETC) and complex V (CV) or ATP synthase. In addition, there are mobile electron carriers such as coenzyme Q (CoQ) and cytochrome c (cyt c), as well as membrane transporters that include among others the pyruvate carrier (not shown), adenine nucleotide translocase (ANT) and the phosphate carrier (PiC). Other FAD-dependent dehydrogenases transfer electrons to CoQ, such as glycerol-3-phosphate dehydrogenase (G3PDH), dihydroorotate dehydrogenase (DHOH), acyl-CoA dehydrogenase complex (ACDH), electron transfer flavoprotein (ETF) and electron transfer flavoprotein dehydrogenase (ETFDH). The electron transfer from NADH and FADH_2_ to molecular oxygen (O_2_), the final electron acceptor, builds a proton-motive force (∆*p*) by the pumping of protons through complexes I, III and IV, which is used by ATP synthase to produce ATP; (**b**) Structure of monomeric bovine active ATP synthase (left) and bound to the N-terminal inhibitory fragment of IF1 in the inhibited ATP synthase state (right). The soluble F_1_ domain is composed of *α_3_β_3_* subunits (purple/blue) and the central stalk (*γ* subunit, light blue, and *δ*, *ε* subunits, light yellow), while the F_o_ domain is formed by a ring of 8 *c* subunits (pink) and *a* subunit (light blue). These two domains are linked by a peripheral stalk, composed of *b*, *d*, *F6* subunits (light green), and the oligomycin sensitivity-conferring protein (OSCP; grey). The supernumerary subunits include *e* (orange), *f*, *g*, A6L, the protein associated with diabetes in insulin-sensitive tissues (DAPIT), and the 6.8 kDa proteolipid (6.8PL) (light blue). Right, the interaction between the N-terminal inhibitory fragment of IF1 (red) and the *α_3_β_3_* and *γ* subunits is shown. The Ala14 residue of IF1 (Ser14 in human and mouse IF1) is highlighted in yellow. The active ATP synthase is able to synthetize ATP using ADP and Pi. When IF1 is bound it is inhibited and no longer synthetizes nor hydrolyzes ATP. Regulation of the ATP synthase is exerted by IF1. IF1 can be inactivated by forming oligomers that mask the inhibitory regions that bind to the F_1_ domain when the matrix pH is above neutrality. However, in mitochondrial de-energization conditions the pH drops below neutrality and IF1 becomes activated. On the other hand, IF1 can be phosphorylated in S39 by PKA preventing its interaction with the ATP synthase. IF1 has a short-half life and there is no evidence yet for the existence of an IF1 protein phosphatase (PPase). Molecular reconstruction from PDB: 6ZPO, 1OHH and 1GMJ. Images created using the PyMOL molecular graphics system.

The OXPHOS system integrates the complexes of the electron transport chain (ETC) (complex I–IV), a variety of different dehydrogenases, the ATP synthase, two diffusible electron carriers such as CoQ and cytochrome c, and substrate transporters in the inner mitochondrial membrane (IMM) (Figure 2a) [30,67,68]. Respiratory complexes of the ETC, and a variety of FAD-dependent dehydrogenases that feed electrons directly to CoQ, transfer electrons obtained in biological oxidations in the form of NADH and FADH_2_ down to molecular oxygen, to generate the proton electrochemical gradient responsible for the proton-motive force (∆*p*) across the IMM (Figure 2a). The ATP synthase utilizes ∆*p* by coupling the backflow of protons into the matrix of the organelle for the generation of ATP from ADP and inorganic phosphate (Pi) (Figure 2a).

The ATP synthase, a protein complex composed of 18 different proteins in humans, drives the production of ATP using the free energy stored in the proton-motive force (Figure 2a,b) [69]. It consists of two main domains, the F_o_ embedded in the IMM and the F_1_ domain projected into the matrix, containing the *α* and *β* subunits of the catalytic core (Figure 2b) [70]. The F_o_ and F_1_ domains are linked together by a central (subunit *γ*, *ε* and *δ*) and a peripheral (subunit *b*, *d*, OSCP and F6) stalk (Figure 2b). The protons are imported into the matrix through subunit a causing the rotation of the *c*-ring in the F_o_ domain. The central stalk transfers this rotational energy to the *α_3_β_3_* subunits in the F_1_ domain, leading to the synthesis of ATP (Figure 2b). The majority of ATP synthase subunits are encoded in nuclear genes with the exception of two hydrophobic subunits (subunit *a* and *8* or A6L) that are encoded in the mitochondrial genome [70]. However, the ATP synthase is a reversible engine. In situations where the ∆*p* collapses, such as hypoxia/anoxia, the ATP synthase acts in reverse to maintain the ∆*p* at the expense of hydrolyzing ATP.

The ATP synthase has a small inhibitory protein, the ATPase Inhibitory Factor 1 (IF1), that for long time has been considered an inhibitor only (unidirectional) of the hydrolytic activity of the enzyme by binding the *αβ* interface in the F_1_ domain (Figure 2b) [71,72]. IF1 is a highly conserved protein among mammalian species and is encoded in the nuclear *ATP5IF1* gene [73]. In the last decade, we and others have demonstrated that IF1 not only inhibits the ATPase activity of the enzyme, but also inhibits its ATP synthetic activity [74,75,76,77,78], promoting the re-emergence of IF1 as the natural physiological inhibitor of both ATP synthase activities, mostly by results in genetic mouse models of loss and gain of function of *Atp5if1* [79,80,81,82,83,84].

**Figure 3:**
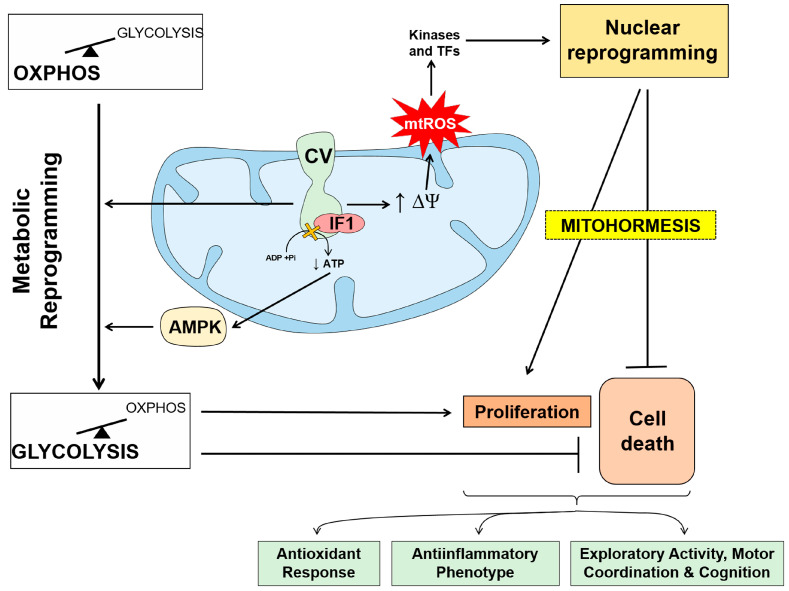
The role of ATP synthase/IF1 axis in metabolic reprogramming and signaling. Binding of IF1 to the ATP synthase limits the production of ATP and promotes metabolic reprogramming towards an enhanced glycolytic phenotype by allosteric regulation of the enzymes of glycolysis and the activation of AMPK. Inhibition of ATP synthase prevents the backflow of protons into the matrix increasing the mitochondrial membrane potential (∆Ψm). The increase in ∆Ψm leads to an increase in the generation of mitochondrial reactive oxygen species (mtROS) at the electron transport chain that signal to the nucleus through kinases and transcription factors. Nuclear reprogramming promotes the activation of cellular responses that include proliferation, prevention of cell death allowing adaptation of the cell to changing cues in mitohormetic responses. The cellular signaling response mediated by IF1 overexpression is cell type specific in mouse tissues and promotes antioxidant responses, anti-inflammatory phenotypes and motor coordination and cognition. In contrast, IF1 overexpression in skeletal muscle and heart is deleterious.

The IF1-mediated inhibition of ATP synthase results in partial blockade of the import of protons into mitochondria, the limitation of OXPHOS activity and the metabolic reprogramming of the cells to an enhanced glycolysis (Figure 3) [74,75,84,85]. Moreover, the blockage in proton import also promotes mitochondrial hyperpolarization and the subsequent production of ROS (mtROS) at respiratory complexes of the ETC (Figure 3) [75,82]. The generated mtROS modify the activity of protein kinases and of transcription factors that signal to the nucleus different programs that allow adaptation of the cell to changing cues (Figure 3) [75,79,80,81,82]. In some mouse tissues, the IF1-mediated activation of mtROS signaling promotes long-lasting metabolic and molecular cytoprotective mechanisms that allow cells to withstand subsequent insults (Figure 3) [79,80,81], a process known as mitohormesis [1,2,3,86]. However, in other mouse tissues which are naturally devoid of IF1 [87], the overexpression of IF1 is detrimental [80,88,89]. In sharp contrast, the overexpression of IF1 in neurons protects from excitotoxic insults [79] and increases the exploratory activity, motor coordination and cognition of mice (Figure 3) [82]. Similarly, the overexpression of IF1 in intestinal epithelial cells provides protection against inflammation (Figure 3) [81], whereas IF1 ablation results in a pro-inflammatory phenotype of fatal consequences [83]. Overall, these results emphasized the relevance of the ATP synthase/IF1 axis in signaling and controlling tissue-specific and non-cell autonomous cell fate decisions.

**Figure 4:**
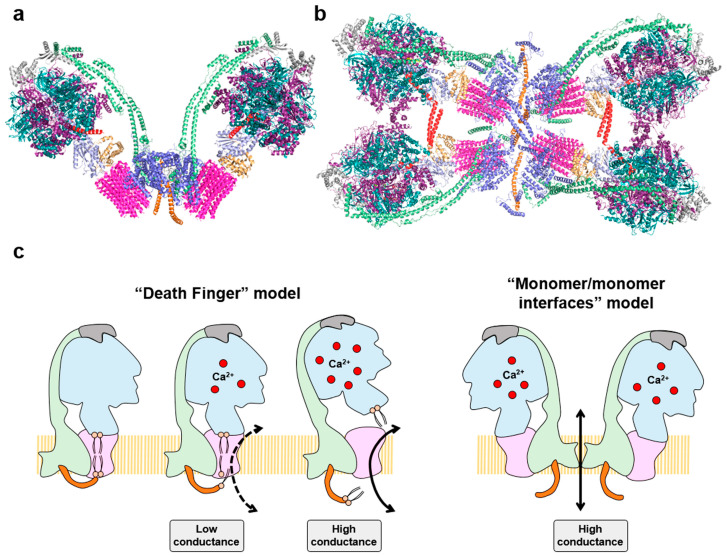
Oligomeric assemblies of the ATP synthase and the permeability transition pore (PTP): (**a**) Structure of bovine dimeric ATP synthase promotes the curvature of the inner mitochondrial membrane at its tips. Molecular reconstruction from PDB: 7AJD; (**b**) Cryo-electron microscopy structure of porcine IF1-inhibited ATP synthase tetramers viewed from the matrix side. The IF1 dimers (red) bind to two adjacent antiparallel ATP synthase dimers. Molecular reconstruction from PDB: 6J5K. Images created using the PyMOL molecular graphics system. Both in a, and b, the color coding of the subunits is the same as in Figure 2; (**c**) Two major models explain the participation of the ATP synthase in the PTP. The “death finger” model proposes that Ca^2+^ binding to *β* subunit causes a rearrangement in the F_1_ domain increasing its rigidity that is transmitted through OSCP (grey) to the peripheral stalk (green) and to the *e* subunit (orange). Subunit *e* exerts a pulling force on the outer lipids that plug the center of the *c*-ring, leading to the formation of a channel that allows the entrance of ions. Mild Ca^2+^ levels lead to closed and open states in a low-conductance mode of opening (“flickering”) in the PTP. However, when Ca^2+^ increases the channel is more prone to opening, promoting complete displacement of inner lipids in the *c*-rotor and of the central stalk of the ATP synthase, committing cells to death by the high-conductance PTP. In the “monomer/monomer interfaces” model, the Ca^2+^ induced conformational changes in dimers of the ATP synthase destabilize the dimeric assembly opening a high conductance pore in the interface of the dimer (subunits *e* and *g*).

Besides its function in energy provision and in signaling, the ATP synthase also plays a structural role in the IMM by forming dimers and oligomers to shape cristae at its rims (Figure 4a,b) [70]. Recent cryo-EM structures of mammalian tetrameric ATP synthases (Figure 4b) [90,91] and isobaric quantitative Protein Interaction Reporter (iqPIR) cross-linking technologies [84], have uncovered the structural role that IF1 plays in oligomerization and inhibition of ATP synthase, and in reprogramming of the tissue to an enhanced glycolysis [84]. Indeed, the overexpression of IF1 in forebrain neurons [82] and cardiomyocytes [84] or of the active mutant version of IF1 (H49K) in liver [80] and skeletal muscle [89] of transgenic mice promotes the oligomerization and inhibition of ATP synthase.

Moreover, the ATP synthase is also a critical component in the regulation and execution of cell death since its components [92] and activity [93,94,95] are required for the efficient execution of cell death. In fact, the ATP synthase is structurally and functionally implicated in permeability transition (mPT) [96,97,98]. mPT is exerted through a high-conductance channel in the IMM, known as the Permeability Transition Pore (PTP) [99,100,101]. PTP opening leads to mitochondrial swelling and cell death and is regulated by Ca^2+^ and/or cyclophilin D-dependent structural changes on ATP synthase [97,102], whereas it is inhibited by cyclosporine A (CsA) [103,104]. The ATP synthase is postulated as a key component of the PTP [96,97,105,106,107,108], although its participation in mPT has been questioned [109,110]. Nowadays, the participation of the ATP synthase in PTP is undisputed after the reunification of the different alternatives that implicated the enzyme in the “death finger” and “monomer/monomer interfaces” models of the PTP (Figure 4c) [96,97,98,102]. In both models, when Ca^2+^ binds the *β* subunit it causes a rearrangement in the F_1_ domain increasing its rigidity [111], that is transmitted through OSCP, a subunit placed on top of the F_1_ domain, to the peripheral stalk and to the *e* subunit (Figure 4c). Because of this structural change, in the “death finger” model subunit *e* exerts a pulling force on the outer lipids that plug the center of the *c*-ring, leading to the formation of a channel that allows the entrance of ions (Figure 4c) [91,96,97,98]. Under physiological levels of matrix Ca^2+^, the PTP oscillates between closed and open states in a low-conductance mode of channel opening, representing the “flickering” modality of the PTP (Figure 4c) [96,97]. However, as matrix Ca^2+^ increases, the channel is more prone to openings, promoting complete displacement of inner lipids in the *c*-rotor and of the central stalk of the ATP synthase, committing cells to death by the high-conductance PTP (Figure 4c) [97,98]. In the alternative “monomer/monomer interfaces” model, the Ca^2+^-induced conformational changes trigger partial changes in the monomer-monomer interfaces (*e*/*g* subunits) of ATP synthase dimers, leading to the formation of the high-conductance PTP (Figure 4c) [97,98].

Interestingly, we and others have demonstrated that the expression of IF1 prevents cell death [75,112,113] and promotes in vivo the oligomerization of ATP synthase in mouse livers, hearts, skeletal muscles and neurons [80,82,84,89], in agreement with cryo-EM studies that show that dimers of IF1 bind two antiparallel dimers of the ATP synthase in a tetrameric inhibited structure of the enzyme (Figure 4b) [90,91]. Hence, it is tempting to suggest that the IF1-mediated oligomerization of the ATP synthase prevents the Ca^2+^-induced conformational changes in the enzyme that result in PTP opening and cell death. In fact, recent findings in isolated mitochondria of intestinal epithelial cells of IF1-ablated mice confirmed that IF1 is necessary to oligomerize and inhibit a fraction of ATP synthase [83]. This mechanism confers resistance to the Ca^2+^-induced PTP opening [83], supporting the theory that IF1-mediated oligomeric states of the ATP synthase act as a structural brake to prevent cell death.

## 4. ATP Synthase and the Bioenergetic Signature of Cancer

As previously mentioned, the high rates of pyruvate fermentation observed in cancer cells under aerobic conditions lead Warburg to suggest that carcinomas had an impaired bioenergetic activity of mitochondria [114]. However, while the upregulation of glycolysis in carcinomas is nowadays out of the question, the alteration of mitochondrial bioenergetics is still a matter of debate, despite the obvious evidence linking the down-regulation of mitochondrial metabolism and bioenergetics in cancer progression [20,35,51,115,116,117,118,119,120]. Perhaps the debate emerges due to the metabolic behavior of some cancer cells when growing in culture [121], differences in the mitochondrial proteome and activity in different tissues [122,123], the heterogeneity of the microenvironment of carcinomas [124,125,126,127,128] and/or the finding that metastatic disease requires an enhanced expression of OXPHOS proteins [129,130,131,132,133,134,135]. In any case, mitochondrial structure and its proteomic composition is certainly altered in a large number of carcinomas [115,136,137,138,139] and, as recently reviewed [35], many of the mitochondrial proteins involved in OXPHOS and in other mitochondrial activities remain unaltered or inhibited when compared to their non-tumor adjacent tissues (NAT). In general, the loss of expression of mitochondrial proteins correlates with a bad prognosis for the patients [20,35]. Overall, these results suggest that OXPHOS is diminished, especially when compared to the sharp induction of glycolysis and other metabolic pathways (Figure 1). In this scenario, the ATP synthase, that integrates the bioenergetic and death-signaling functions of mitochondria, is called to play a relevant function in cancer onset and progression.

Indeed, the down-regulation of the catalytic subunit of mitochondrial ATP synthase (β-F1-ATPase) is observed in a large number of human carcinomas [20,35,115,140], either when expressed in absolute levels or normalized relative to other mitochondrial proteins, such as Hsp60 and/or to the glycolytic GAPDH. In fact, the quotient of the expression level of these three biomarkers β-F1-ATPase/Hsp60/GAPDH offers a bioenergetic cellular (BEC) index that informs about the overall capacity of mitochondria in the tissue or carcinoma [26,115]. Moreover, the BEC index is essentially the same in lung, breast and esophageal carcinomas despite the large differences in the expression of these proteins in the corresponding non-tumor adjacent tissues (NAT) [141]. These findings suggest that transformations blur the tissue-specific differences in energy metabolism to converge on the same metabolic phenotype that sustains tumor growth. Interestingly, a low expression of β-F1-ATPase and a low BEC index in the carcinoma predicts poor overall (OS) and disease free survival (DFS) for colon cancer patients [115], suggesting that the downregulation of OXPHOS contributes to tumor progression [26,115,142]. The relevance of a low BEC index in predicting a poor prognosis for patients bearing different types of carcinomas (breast, colon, lung, melanoma, ovarian, gallbladder) and leukemias has been extensively documented [16,35,39,115,142,143,144,145,146,147,148,149,150]. Furthermore, the BEC index significantly correlates with migration speed and in predicting drug responses across the NCI-60 cell line panel [151], as well as in predicting the response to different targeted therapies in preclinical mouse models bearing different human carcinomas [20,39,51,60,61,62,120,152,153].

Remarkably, a high-throughput quantitative analysis of 27 biomarkers of metabolism—using Reverse Phase Protein Array (RPPA) technology, in a cohort of 128 tumors and the corresponding paired NAT of patients bearing lung adenocarcinomas (LUAD)— confirmed that the BEC index is significantly diminished in the carcinomas, suggesting that OXPHOS activity is limited in LUAD [20], in agreement with previous reports [16,23,145]. In fact, discrimination of a tumor from NAT was accomplished by the signature of six metabolic proteins analyzed [20]. Remarkably, five out of the six proteins in the signature are mitochondrial proteins, strongly suggesting that the mitochondrial proteome is a most relevant target in metabolic reprogramming of LUAD [20].

Interestingly, and despite the content of β-F1-ATPase is not increased in the whole cohort of LUAD studied [20], the analysis revealed that the subgroup of patients bearing carcinomas with the highest content of β-F1-ATPase had a worse prognosis when compared to the low content subgroup of patients [20], in agreement with a previous report [66]. Similar findings have been reported in large cohorts of breast [144] and melanoma [146] patients suggesting that, despite there being an overall limitation of OXPHOS in the carcinomas, mitochondrial bioenergetics is required for metastatic disease and cancer progression (see below) [131,154,155].

The downregulation of the mitochondrial ATP synthase in cancer can be mediated at the expression and activity levels [30]. The expression of the catalytic subunit of ATP synthase (β-F1-ATPase) in mammalian liver is complex, including the subcellular localization of its mRNA in a structure that is frequently found attached to the outer mitochondrial membrane [156,157]. β-F1-ATPase mRNA localization and translation requires at least two cis-acting elements and a large set of RNABPs [156,158] and the 3′-UTR of the mRNA that acts as a translational enhancer sequence [159,160,161,162]. Consistently, the expression of β-F1-ATPase in prevalent human carcinomas, in differentiation and during the cell cycle [51,159,163,164,165,166,167], is also controlled at the level of translation by a set of mRNA-binding proteins that upon binding the 3′UTR of β-F1-ATPase mRNA inhibit its translation [30,160,166]. Remarkably, some of the mechanisms of translational control of β-F1-ATPase are conserved between liver hepatocarcinogenesis [168] and rat liver development [159,163,169].

An affinity purification approach of RNABPs using as bait the full-length β-F1-ATPase mRNA allowed the identification of nine bona fide mRNA binding proteins [170]. Among them, G3BP1 (Ras-GTPase-Activating Protein SH3-Domain-Binding Protein) was found to interact both in vivo and in vitro with the 3′UTR of β-F1-ATPase mRNA, to promote inhibition of the synthesis of the protein by preventing mRNA translation [30,166,170]. Interestingly, G3BP1 is overexpressed in breast carcinomas [171] and its overexpression predicts shorter time for recurrence of the disease [30]. Likewise, G3BP1 is also overexpressed in gastric cancer representing an independent prognostic factor of poor prognosis [172]. These results stress the relevance that translational control exerted by RNABPs has in the biogenesis of ATP synthase, and hence, in the bioenergetic signature and function of mitochondria.

In addition, translational control of β-F1-ATPase mRNA (β-mRNA) is also exerted by miR-127-5p that targets the 3′UTR of the mRNA and significantly reduces its translational efficiency without affecting β-mRNA abundance [173]. Interestingly, this mechanism of translational control for the expression of mitochondrial ATP synthase is operative in fetal human and rat livers but not in oncogenesis [173].

Furthermore, both in Acute Myeloid Leukemia and Chronic Myeloid Leukemia the downregulated expression of β-F1-ATPase is exerted by hypermethylation of the promoter of the *ATP5F1B* gene, limiting the availability of β-F1-ATPase mRNA in cancer cells [150,174]. Overall, these findings just uncover the “tip of an iceberg” that represents the scarcely studied field of translational control in regulating the biogenesis of mitochondria and of the ATP synthase, which has paramount implications in the tissue-specific biogenesis of the organelle and in cancer progression.

## 5. ATPase Inhibitory Factor 1, the Physiological Inhibitor of the ATP Synthase

As mentioned above, the control of the overall ATP synthase activity is not only exerted at the protein level, but also by regulation of the content and activity of IF1, its inhibitor protein. For many years, it has been considered that under normal physiological situations, i.e., when the mitochondrial matrix pH is above neutrality, IF1 forms inactive oligomers by masking the inhibitory regions that bind to the F_1_ domain of the enzyme (Figure 2b) [175,176]. Only when mitochondria become de-energized, and matrix pH drops below neutrality, IF1 is considered to become activated by depolymerization and binds to the ATP synthase to inhibit its hydrolase activity (Figure 2b) [175,176]. pH regulation of IF1 activity involves protonation/deprotonation of five conserved histidine residues that play a relevant role in structural changes and oligomerization [175,177]. Interestingly, the IF1-H49K mutant retains its inhibitory activity on the enzyme even at pHs above 6.7, both in vitro [74,85,175,177] and in vivo under basal physiological conditions of mitochondria [79,80,89].

Our studies in metabolic reprograming in human carcinomas revealed that normal lung, colon and breast tissues express negligible quantities of IF1 [85] but showed a sharp increase in the expression of the protein in the carcinomas and in cultured cancer cells [74,75,85]. These findings raised the reasonable hypothesis that IF1 might represent a sort of mitochondrial “oncogene” [178], not only to control the hydrolytic activity of the ATP synthase, as it was postulated many years ago, but also its ATP synthetic activity. In other words, the overexpression of IF1 could be mediating the partial inhibition of OXPHOS contributing to the metabolic reprogramming experienced in oncogenesis. Indeed, the overexpression of IF1 in several cancer cells [74,75,77,78,85], mouse models of gain of function of IF1 [79,80,81,82,84,89] and pharmacologic in vivo approaches [76], amply demonstrated that IF1 overexpression inhibits the ATP synthetic activity of ATP synthase in isolated mitochondria, diminished tissue ATP levels, activated AMPK and promoted the metabolic rewiring of cells and tissues to an enhanced glycolytic phenotype, i.e., induced the Warburg phenotype. Conversely, silencing or knocking out IF1 in cancer cells [20,179] and mouse models of loss of function of IF1 [82,83] resulted in and enhanced activity of the ATP synthase.

Stem cells, iPSC and cancer cells have a predominant glycolytic phenotype whereas OXPHOS prevails in differentiated cells [180,181]. Interestingly, IF1 also plays a relevant role in metabolic reprogramming during differentiation of human mesenchymal stem cells (hMSC) and in the maintenance/acquisition of stemness [182]. In fact, hMSC also present high levels of IF1, altered mitochondrial structure and molecular composition, low rates of OXPHOS and an enhanced glycolytic metabolism [182]. However, when hMSC cells are induced to differentiate into osteocytes, the expression of IF1 is lost by an enhanced rate of degradation of the protein that is concurrent with the biogenesis of OXPHOS proteins, the increase in oxygen consumption rates and the partial inhibition of glycolysis [181,182]. On the contrary, nuclear reprogramming of somatic cells to induce pluripotency (iPSC) promotes the downregulation of OXPHOS concurrently with the activation of glycolysis [183] and the re-expression of high cellular levels of IF1 [184]. Altogether, these findings emphasize the relevance of IF1 in metabolic reprogramming in cancer and pluripotent stem cells.

Remarkably, the results in tissues of mouse models with regulated expression of IF1 further suggest that mitochondria in non-stressed physiological conditions of some cellular types such as cardiomyocytes [76], neurons [82] and intestinal epithelial cells [83] contain a fraction of IF1-bound and -inhibited ATP synthase, in agreement with recent cryo-EM studies of mammalian heart ATP synthases [90,91]. We suggest that the IF1-inhibited ATP synthase in these tissues, besides regulating the output of ATP as a function of energy demand, is involved in controlling mitochondrial signaling, structure and cell death as discussed in a previous section.

The regulation of IF1 activity as an inhibitor of ATP synthase is also exerted by covalent modification of the protein by phosphorylation (Figure 2b). Phosphoproteomic studies indicated that serine residues of IF1 are phosphorylated in cancer cells [185,186,187]. Indeed, we showed that IF1 is phosphorylated in several serine residues in cells in cultures and in mouse hearts in vivo in response to cAMP-dependent activation of PKA [76]. Site-specific phosphodeficient and phosphomimetic IF1 serine mutants revealed that the PKA-mediated phosphorylation of IF1 in S39, but not in other serine residues, prevented the interaction of IF1 with ATP synthase and enhanced both the ATP synthetic and hydrolytic activities of the enzyme (Figure 2b) [76]. Interestingly, it should be noted that tissues that contain high expression levels of IF1 present both phospho- and dephosphorylated IF1 [76,87], consistent with the existence of two pools of ATP synthase in their mitochondria, active and IF1-bound inhibited enzyme [76].

Remarkably, recent findings have demonstrated that the binding of kynurenic acid to the GPR35 orphan receptor prevents the phosphorylation of IF1 in human and mouse cardiomyocytes to prevent ATP hydrolysis as an anti-ischemic ATP-conservation mechanism [188,189]. Interestingly, these authors further confirmed that dephosphorylated IF1 binds to ATP synthase and promotes enzyme oligomerization [188], in agreement with recent cryo-EM structures of the oligomeric inhibited enzyme [84,90,91] and our results in mouse models expressing IF1 [79,80,81,82,83,89]. These results further suggest that the content of dephospho-IF1 controls the fraction of oligomeric and inhibited ATP synthase and hence, that the phosphorylation/dephosphorylation of IF1 represents a most relevant mechanism for a fine and rapid adjustment of the function of the OXPHOS system in different physiological situations [73,76].

**Figure 5:**
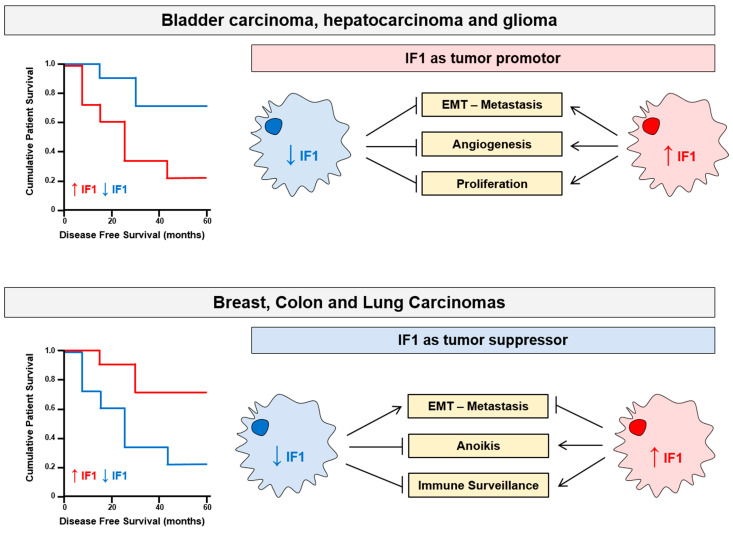
The role of IF1 as tumor promotor or tumor suppressor. In bladder carcinomas, hepatocarcinomas and gliomas, high expression levels of IF1 (red) predict a poor prognosis for the patients when compared to carcinomas with low levels of IF1 (blue). In these carcinomas, IF1 acts as a tissue-specific tumor promotor facilitating proliferation, angiogenesis, epithelial mesenchymal transition (EMT) and metastasis. In contrast, in breast, colon and lung carcinomas, high expression levels of IF1 (red) predict a good prognosis for the patients when compared to carcinomas with low levels of IF1 (blue). In these carcinomas, IF1 acts as a tissue-specific tumor suppressor preventing epithelial mesenchymal transition (EMT) and metastasis by favoring cell death upon cellular detachment and immune surveillance.

Indeed, the phosphorylation of IF1 has been observed when there is an increase in energy demand in the heart in vivo or in cells in cultures when they are forced to increase OXPHOS [76]. Likewise, IF1 is phosphorylated when cells progress through the OXPHOS-dependent G1-phase of the cell cycle [76]. In contrast, hypoxia and progression through the reductive phases of the cell cycle (S/G2/M) promote dephosphorylation of IF1, the reduction of OXPHOS and an enhanced glycolytic flux [76]. Consistently, dephosphorylated IF1 is the prevalent form of the inhibitor protein in lung, colon, and breast carcinomas, contributing to the inhibition of ATP synthase and the reprogramming of metabolism to an enhanced glycolysis and fermentation [76]. As previously mentioned, and besides controlling energy metabolism, the fraction of IF1-bound inhibited ATP synthase in the carcinomas is also fulfilling additional functions through signaling a pro-oncogenic phenotype, by stimulating proliferation/invasion/metastasis (Figure 5) [75,80,88,190,191], or an anti-oncogenic phenotype, by preventing metastatic disease and immune surveillance (Figure 5) (see next section) [20,85,147,179,192].

Besides the mechanisms that regulate the fraction of IF1 that is active as an inhibitor of the ATP synthase discussed above (Figure 2b), the mechanisms that control the overall mitochondrial content of IF1 are also of relevance. As reviewed elsewhere [73], the *ATP5IF1* gene interacts with c-Myc, c-Fos, NFκB and HIF-1α transcription factors. However, it is remarkable that in the early phase of cell reprogramming of somatic cells into pluripotency, c-Myc is the transcription factor that triggers a sharp increase in IF1 expression, precipitating the metabolic program necessary for cellular reprogramming [184].

Most available evidence suggests that regulation of IF1 expression is exerted at post-transcriptional levels by controlling the rates of its synthesis and degradation [182]. In fact, the increase in protein content of IF1 in carcinomas [85] or its sharp reduction during cellular differentiation [182], are exerted in the absence of relevant changes in the abundance of IF1-mRNA. In fact, the IF1 protein has a very high turnover rate (2–3 h), both in cancer cells [85] and in differentiated osteocytes [182], which is much faster than the turnover of many other subunits of ATP synthase in cancer cells (18 h) [193]. IF1 degradation is mediated by several mitochondrial serine proteases [85,182] and metalloproteases [194], but the specific genes involved have not been identified yet [182].

In addition, IF1-mRNA is also subjected to tissue-specific translation control by RNABPs [30,195]. In fact, both in the heart [196] and in the liver [197] knockdown of LRPPRC (leucine rich pentatricopeptide repeat containing protein), a RNABP that is involved in post-transcriptional regulation of mtDNA expression [198], severely reduces the activity and assembly of ATP synthase. In the heart, the inhibition an assembly deficit of ATP synthase is associated with the overexpression of IF1 [196]. Interestingly, LRPPRC specifically interacts with IF1-mRNA [30] and its over-expression in human and mouse cells significantly diminished the expression of IF1, increasing the rate of cellular ATP production [87]. Contrariwise, the silencing of LRPPRC in human and mouse cells augmented IF1 expression and reduced the ATP synthesis rate of the cells [87]. Interestingly, LRPPRC is tissue-specifically expressed [87] and the studies of its relevance in IF1-mRNA translation certainly deserves further investigations that will contribute to uncovering the tissue-specific mechanism that controls the activity of ATP synthase.

## 6. Tissue-Specific Activity of IF1: Tumor Promotor and Tumor Suppressor

A fundamental characteristic of IF1 is that it is a tissue-specific and species-specific expressed protein in mouse and human tissues [87]. Human normal tissues such as the heart, brain, kidney, stomach, endometrium and liver have a high IF1 content, whereas breast, colon, and lung epithelia contain negligible levels of IF1 [85,87]. Likewise, in human carcinomas the expression of IF1 is also variable [85]. Whereas oncogenesis in epithelial cells of the endometrium, stomach and kidney does not promote an increase in IF1 expression, carcinomas in the colon, lung and breast show a very sharp increase in the content of IF1 [85].

In agreement with the pro-oncogenic role of IF1, studies in human hepatocellular carcinomas (HCC) showed that high levels of IF1 predict worse overall and progression free survival for the patients (Figure 5) [88]. Mechanistically, IF1 activates the non-canonical NFκB pathway, driving angiogenesis and metastasis through the activation of SNAI1 and VEGF [88]. Consistent with these findings, transgenic mice overexpressing in the liver the constitutively active mutant IF1-H49K promoted the inhibition of OXPHOS and a state of metabolic preconditioning guided by the activation of the stress kinases AMPK and p38 MAPK [80]. Diethylnitrosamine-induced liver carcinogenesis in IF1-H49K transgenic mice contributed to an enhanced liver carcinogenesis by augmenting proliferation and apoptotic resistance of carcinoma cells [80]. Likewise, the overexpression of IF1 in bladder carcinomas [190] and in gliomas [191] also predict a worse prognosis for the patients and a shorter time to disease recurrence (Figure 5). Mechanistically, the silencing of IF1 diminished the rates of proliferation, migration and invasive capacities of the cells, preventing epithelial mesenchymal transition (EMT) (Figure 5) [88,190,191]. In contrast, the overexpression of IF1 in these cancer cells promoted angiogenesis and activation of EMT, increasing the expression of E-cadherin and diminishing that of vimentin, to promote their metastatic capacity (Figure 5) [88,190,191].

However, in breast [85,147], colon [85,179] and lung [20] carcinomas, high levels of IF1 expression correlate with a good prognosis for the patients (Figure 5), suggesting that in these tissues the expression of IF1 acts as a tumor suppressor. We have investigated the molecular basis of the tumor-suppressor function of high IF1 expression in breast, colon and lung cancer cells, by means of the generation of stable cancer cell lines that overexpress or are devoid of IF1 [20,147,179]. In these studies, cancer cells overexpressing IF1 restrain OXPHOS, enhance glycolysis and trigger the production of a mtROS signal that activates transcription of the NFκB promoter which results in protecting cells from death-inducing agents [20,75,85,147,179]; in other words, it would initially appear that IF1 is favoring a phenotype prone to cancer progression. However, the overexpression of IF1 in these cancer cells also promotes a transcriptome that indicates a more epithelial phenotype when compared to IF1-ablated or knockdown cells (Figure 5) [20,147,179]. In fact, we showed that IF1 overexpressing cancer cells proliferate, migrate and invade less than cancer cells with low expression levels or that are devoid of IF1 (Figure 5) [20,147,179]. Furthermore, in the case of colon and lung cancer cell lines, the overexpression of IF1 renders the cells prone to anoikis [20,179], the form of cell death upon cellular detachment, presumably because of the limitation of OXPHOS in the metastatic cell. In addition, colon spheroids of IF1-silenced cells have an increased ability to escape from immune surveillance by Natural Killer cells (Figure 5) [179], thus favoring metastasis. As a result, tumor development in mouse xenografts using colon and lung cancer cells confirmed that IF1-silenced or IF1-ablated cells are more tumorigenic and metastatic in vivo than IF1-overexpressing cells [20,179]. Overall, despite that a high IF1 level confers to breast, colon and lung cancer cells a more glycolytic phenotype, it restrains tumor growth and metastatic disease by repressing OXPHOS and thus underpinning the better prognosis of the cancer patients that bear tumors with high expression levels of IF1 (Figure 5). However, it is reasonable to suggest that the tumor suppression function afforded by the mitochondrial dose of IF1 in these types of carcinomas also depends on the nuclear reprogramming exerted by the activation of specific kinases and transcription factors mediated by mtROS signaling generated as a result of the IF1-mediated inhibition of the ATP synthase (Figure 3). In this regard, it should be noted that the activation of these cellular programs is different depending on the cell type as shown in tissue-specific mouse models of loss and gain of function of IF1 [79,80,81,84,89].

**Figure 6:**
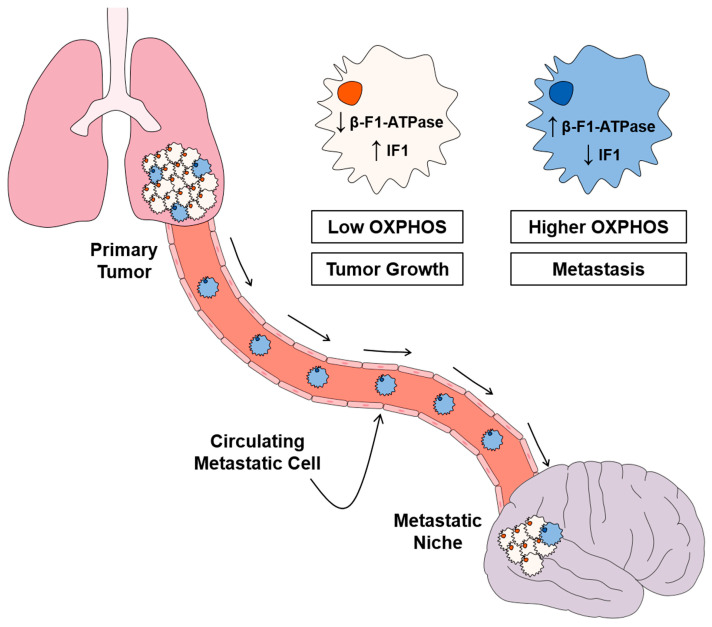
The ATP synthase/IF1 axis in metastatic disease. A working scheme summarizes the interplay of the two biomarkers of OXPHOS (β-F1-ATPase and IF1) involved in the metastatic signature of lung adenocarcinomas [20]. Low and high OXPHOS cells coexist in the primary tumor. Low OXPHOS cells (light orange cells; low of levels of β-F1-ATPase and high levels of IF1) promote the growth of the primary tumor through an enforced glycolysis. Cells with higher OXPHOS (blue cells; high of levels of β-F1-ATPase and low levels of IF1) prime metastatic disease [131]. Metastatic disease requires higher OXPHOS cells because they are less vulnerable to cell death upon cellular detachment from the primary tumor [20,179], and are able to colonize the metastatic niche since they have higher chances to scape immune surveillance [179].

As mentioned above, OXPHOS is required for metastatic disease (Figure 6) [20,129,130,131,133,147,179] and, consistently, melanoma, breast and lung carcinomas [20,66,144,146] with the highest expression level of β-F1-ATPase predict higher chances of disease recurrence for the patients (Figure 6). Interestingly, in the RPPA study of LUAD carcinomas, machine learning provided a highly significant signature of metastasis (*p* = 3.17 10^−19^) that included the expression of β-F1-ATPase, IF1, superoxide dismutase (SOD2), thioredoxin (TRX), peroxiredoxin 6 (PRX6) and 4-hydroxynonenal (4-HNE) modification of proteins [20]. Remarkably, the signature included the two biomarkers of OXPHOS. A high β-F1-ATPase represented a bad independent predictor of OS (HR (95% CI) = 4.524(0.955–21.43), *p* = 0.05) whereas a high IF1 expression level represented a good independent predictor of recurrence of the disease (HR (95% CI) = 0.11(0.025–0.51), *p* = 0.005) (Figure 6) [20]. In agreement with these results, it has been reported that cancer cells with higher invasive capacity experience an increase in mitochondrial respiration as well as ATP production (Figure 6) [131]. Moreover, metastatic breast cancer cells have lower IF1 levels when compared to the primary tumors [199], suggesting that metastatic cells rely on OXPHOS when compared to cancer cells in the primary tumor (Figure 6) [131].

Overall, we believe that therapeutic approaches targeting the ATP synthase/IF1 axis and hence, the activity of OXPHOS in carcinomas, should take into consideration the tissue in which the carcinoma develops.

## 7. Mitochondria: A Promising Target for Cancer Treatment

Novel therapeutic approaches based on personalized medicine are required to minimize the social and economic burden caused by cancer. The enzymes that control metabolism are promising targets to combat progression of the disease [9,14,26,200,201]. In this regard, several glycolytic inhibitors have been proposed and some of them are actually in clinical trials [26,51,152,201,202,203,204]. Mitochondrial metabolism also offers other effective targets to restrain tumor growth [201,203,205,206,207,208]. In this regard, antibiotics such as doxycycline that target the mitochondrial ribosome and inhibit translation prevent mitochondrial biogenesis which is required for the clonal expansion and survival of cancer stem cells (CSCs) [209]. Doxycycline has also been used in combination with other anticancer therapies [210,211,212,213] and their effectiveness lies in their ability to prevent metastasis by inhibiting the growth of CSCs. Interestingly, a Phase II clinical pilot study has demonstrated that doxycycline reduces the expression of stemness markers limiting the CSC burden in early breast cancer patients [214]. Tri-phenyl-phosphonium (TPP)-related compounds also offer novel strategies for cancer treatment, as they inhibit CSC metastasis either as stand-alone [215] or in combination with an inhibitor of mitochondrial translation [216]. Moreover, cyanine dyes also inhibit mitochondrial metabolism suppressing the growth CSCs impeding metastasis in vivo [217]. Other examples are those afforded by metformin [218] and phenformin [219,220] that promote the inhibition of mitochondrial OXPHOS and reduce the risk of cancer in diabetic patients, increasing their rates of survival. More recently, the FDA-approved bedaquiline, which promotes the downregulation of the *γ* subunit of the ATP synthase, inhibits mitochondrial ATP production and metastasis in vivo [221,222]. These results emphasize the importance of drug repurposing strategies to accelerate clinical translation of drugs for treatment of cancer—in other words, the use of drugs already available and with acceptable known side effects in humans to improve the quality of life of cancer patients [223,224].

In this line, we recently found that nebivolol, a third-generation β1-blocker, halts colon and breast tumor growth in vivo by a severe inhibition of OXPHOS [60]. Mechanistically, nebivolol binds to β1-adrenergic receptors in cancer cells, causing the increase in IF1 content bound to the ATP synthase and promoting its inhibition. Concurrently, nebivolol prevents the phosphorylation of NDUFS7, a Complex I subunit, which promotes partial inhibition of the activity of the enzyme [60]. In addition, nebivolol also arrests proliferation of endothelial cells by blocking β1-adrenergic receptors impeding tumor angiogenesis [60]. Hence, nebivolol treatment promotes the arrest of colon, breast, lung and squamous cell carcinomas [20,60,225] by promoting a metabolic and redox crisis in cancer cells [60]. We propose that nebivolol is a very strong candidate to be repurposed in “basket trials”, those in which cancer patients with carcinomas from different origins are included. Specially, to prevent cancer recurrence and metastasis after conventional cancer therapies that render cancer cells OXPHOS-dependent [226,227,228,229].

Moreover, OXPHOS is not the only target in mitochondria to combat cancer. In fact, enzymes of fatty acid oxidation, such as carnitine palmitoyltransferase 1 (CPT1), electron transfer flavoprotein subunit α (ETFA) and hydroxyacyl-CoA dehydrogenase trifunctional multienzyme complex subunit α (HADHA), are increased in LUAD [20], suggesting that the oxidation of fatty acids is required for cancer progression and might provide a target for therapy. Indeed, inhibitors of fatty acid oxidation have been demonstrated to be promising drugs to be repurposed in the case of leukemia cells [230], breast [231] and colon carcinomas [232]. Hence, it is possible to design therapies that simultaneously target two metabolic pathways of mitochondria. Indeed, we have targeted lung adenocarcinomas in mice by restraining the assimilation of fatty acids with orlistat, or its oxidation in mitochondria with etomoxir, in both cases in combination with nebivolol [20]. With this approach, we demonstrated that the combined treatment provided better welfare and extended the lifespan of mice greater than conventional treatment with cisplatin [20]. Overall, these results highlight mitochondria as a gold standard target for the effective treatment of a large diversity of carcinomas.

## 8. Conclusions and Future Directions

Despite major genetic advances in the understanding of cancer, cancer patients still require the development of innovative therapeutic strategies matched to the phenotype of their tumors to halt progression of the disease. The accumulated evidence indicates that the metabolic reprogramming experienced in cancer provides new biomarkers that, alone or in combination, could be exploited to halt the disease in a successful personalized medicine. In this review, we have summarized the different functions that the mitochondrial ATP synthase and its inhibitor protein, IF1, play in cellular biology and in cancer progression. We have overviewed the mechanism by which the ATP synthase/IF1 axis contributes to metabolic reprogramming to an enhanced glycolytic phenotype, both in cancer cells and in the maintenance of stemness, and its potential both as biomarkers of prognosis and as targets for therapy. Moreover, we have highlighted how the ATP synthase/IF1 axis contributes to the signaling of cell-type specific programs that allow the adaptation of the cell/organisms to different changing cues, and finally, how the ATP synthase/IF1 axis also participates in preventing the execution of cell death and hence, in therapeutic resistance of the carcinomas. We have emphasized that the relative low activity of mitochondrial metabolic pathways, such as OXPHOS and β-oxidation in lung adenocarcinomas, contribute to cancer progression. In fact, the analysis of the expression of enzymes of metabolism revealed that the alteration of mitochondria is a most evident target in lung adenocarcinomas, and both the catalytic subunit of ATP synthase (β-F1-ATPase) and IF1, provide two most relevant biomarkers of metastatic disease. Actually, and depending on the type of carcinoma, the expression levels of IF1 define its activity as a tumor suppressor or as an oncogene. Although the glycolytic phenotype is needed for tumor growth, metastatic disease requires the activity of OXPHOS, i.e., relatively high β-F1-ATPase expression in the carcinoma. However, the activity of ATP synthase is counterbalanced by enhanced expression levels of IF1 that exert tumor suppressor activities in some carcinomas. Hence, targeting mitochondrial ATP synthase and IF1 holds great promise for overcoming therapeutic resistance and impeding metastatic disease. We have emphasized the mechanisms that control the expression and activity of β-F1-ATPase and IF1 in cancer, which show similarities with those operating in development and differentiation, to highlight the relevance that post-transcriptional mechanisms play in the regulation of the mitochondrial phenotype of mammalian tissues and carcinomas. Unfortunately, this is a field that is scarcely investigated, but that could illuminate new biomarkers and therapeutic targets for cancer treatment. Finally, we summarize recent preclinical findings of the potential that OXPHOS and β-oxidation have as targets to prevent cancer progression. We think that research focused on the ATP synthase/IF1 axis will accelerate translation of energy metabolism in more effective and personalized cancer therapies.
